# 1-[4-(β-d-Allopyranos­yloxy)benzyl­idene]semicarbazide hemihydrate

**DOI:** 10.1107/S1600536809055664

**Published:** 2010-01-09

**Authors:** Hua-Feng Chen, Xue Bai, Kuan Zhang, Ying Li, Shu-Fan Yin

**Affiliations:** a College of Chemistry, Sichuan University, Chengdu 610064, People’s Republic of China

## Abstract

The mol­ecule of the title compound, C_14_H_19_N_3_O_7_·0.5H_2_O, exhibits an *E* conformation about the C=N double bond. The water mol­ecule possesses crystallographically imposed twofold symmetry. In the crystal structure, the mol­ecules are connected by inter­molecular O—H⋯O and N—H⋯O hydrogen bonds into a three-dimensional network.

## Related literature

For the properties of helicid (systematic name: 4-formyl­phenl-β-d-allopyran­oside), see: Chen *et al.* (1981 ▶); Sha & Mao (1987 ▶). For the synthesis of the title compound, see: Zhu *et al.* (2008 ▶). For related structures, see: Fan *et al.* (2007 ▶); Yang *et al.* (2008 ▶)
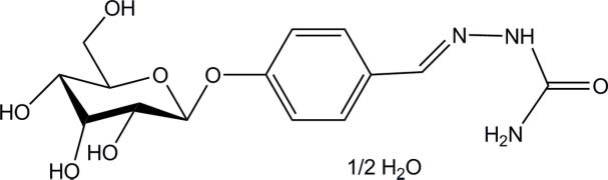


## Experimental

### 

#### Crystal data

C_14_H_19_N_3_O_7_·0.5H_2_O
*M* *_r_* = 350.33
Trigonal, 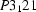

*a* = 8.6373 (12) Å
*c* = 37.021 (7) Å
*V* = 2391.8 (7) Å^3^
*Z* = 6
Mo *K*α radiation
μ = 0.12 mm^−1^
*T* = 113 K
0.28 × 0.25 × 0.21 mm

#### Data collection

Rigaku Saturn CCD area-detector diffractometer
Absorption correction: multi-scan (*CrystalClear*; Rigaku/MSC, 2005 ▶) *T*_min_ = 0.967, *T*_max_ = 0.975
15783 measured reflections
2242 independent reflections
2161 reflections with *I* > 2σ(*I*)
*R*_int_ = 0.038

#### Refinement

*R*[*F* ^2^ > 2σ(*F* ^2^)] = 0.033
*wR*(*F* ^2^) = 0.085
*S* = 1.09
2242 reflections
235 parameters
H atoms treated by a mixture of independent and constrained refinement
Δρ_max_ = 0.45 e Å^−3^
Δρ_min_ = −0.18 e Å^−3^

### 

Data collection: *CrystalClear* (Rigaku/MSC, 2005 ▶); cell refinement: *CrystalClear*; data reduction: *CrystalClear*; program(s) used to solve structure: *SHELXS97* (Sheldrick, 2008 ▶); program(s) used to refine structure: *SHELXL97* (Sheldrick, 2008 ▶); molecular graphics: *ORTEP-3 for Windows* (Farrugia, 1997 ▶); software used to prepare material for publication: *SHELXL97*.

## Supplementary Material

Crystal structure: contains datablocks global, I. DOI: 10.1107/S1600536809055664/rz2403sup1.cif

Structure factors: contains datablocks I. DOI: 10.1107/S1600536809055664/rz2403Isup2.hkl

Additional supplementary materials:  crystallographic information; 3D view; checkCIF report

## Figures and Tables

**Table 1:** Hydrogen-bond geometry (Å, °)

*D*—H⋯*A*	*D*—H	H⋯*A*	*D*⋯*A*	*D*—H⋯*A*
O2—H2⋯O7^i^	0.84	1.85	2.6905 (19)	175
O3—H3⋯O8^ii^	0.84	1.94	2.7447 (16)	159
O4—H4⋯O5^iii^	0.84	2.05	2.8277 (19)	154
O4—H4⋯O5	0.84	2.34	2.7725 (19)	113
O5—H5⋯O2^iv^	0.84	1.85	2.693 (2)	178
N3—H3*A*⋯O4^v^	0.88	2.24	3.096 (2)	165
N3—H3*A*⋯O3^v^	0.88	2.46	2.896 (2)	111
O8—H8*O*⋯O7^vi^	0.84 (3)	1.95 (3)	2.7163 (17)	151 (4)
N2—H2*N*⋯O3^vii^	0.88 (3)	2.14 (3)	3.014 (2)	176 (2)

Symmetry codes: (i) 
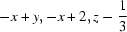
; (ii) 
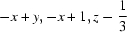
; (iii) 
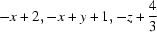
; (iv) 
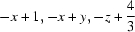
; (v) 
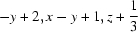
; (vi) 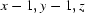; (vii) 
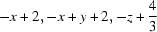
.

**Fig. 1.**
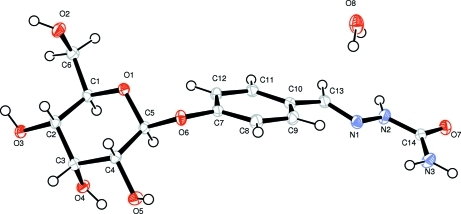
The molecular structure of the title compound, with displacement ellipsoids drawn at the 30% probability level. The water H atoms are related by the symmetry operation 1+x-y, 2-y, 5/3-z.

## supplementary crystallographic information

### Comment

The natural product helicid, 4-formylphenl-β-D-allopyranoside, which is a major active ingredient of herbal medicine, is extracted from the fruit of Helicia nilagirica Beed growing at the Yunnan mountain of China (Chen *et al.*, 1981). It has showed great biological effects on the central nervous system with low toxicity (Sha & Mao, 1987), and has been used to treating ache and insomnia for a long time. Some derivatives of this compound have been reported with good pharmacological activity (Fan *et al.*, 2007; Yang *et al.*, 2008). The title compound has been synthetized *via* condensation reaction of helicid and semicarbazide hydrochloride in ethanol (Zhu *et al.*, 2008).

In this title compound (Fig. 1), the pyranoside ring adopts a stable chair conformation with the hydroxyl group at C3 in axial position and the other substituents at C1, C2 and C4 in equatorial position. The N1═C13 double bond in the molecule exhibits an *E* conformation, as indicated by the values of the C(13)–N(1)–N(2)–C(14) and C(11)–C(10)–C(13)–N(1) torsion angles of 176.28(0.19) and -160.45 (1/5)°, respectively. The average C–C bond lengths in the pyranoside ring is 1.52 (3) Å. The average C(*sp*^3^)–O and C(*sp*^2^)–O bond lengths are 1.426 (2) and 1.319 (2)Å, respectively. Intermolecular O—H···.O and N—H···.O hydrogen bonds (Table 1) are present in the crystal structure, generating a three-dimensional network.

### Experimental

Sodium acetate was added to a solution of semicarbazide hydrochloride (3.52 mmol) in water (5 ml) until pH 5–6. The solution was added to a solution of helicid (3.52 mmol) in ethanol (40 ml), then some drops of glacial acetic acid were added and the mixture was refluxed for 5 h. The reaction mixture was then cooled at room temperature. The colourless solid obtained was filtered, washed with water and recrystallized from ethanol/water (Zhu *et al.*, 2008). Colourless crystals suitable for X-ray analysis were obtained by slow evaporation of an ethanol/water (5:1 *v*/*v*) solution at room temperature.

### Refinement

The independent water H atom and the H atom bound to N2 were located in a difference Fourier map and refined freely. All other H atoms are positioned geometrically and refined in riding mode, with C—H = 0.95–1.00 Å, O—H = 0.84 Å and N—H = 0.88 Å, and with *U*_iso_(H) = 1.2 *U*_eq_ (C, N, O). In the absence of significant anomalous dispersion effects, Friedel pairs were merged. The choice of space group *P*3_1_21 rather than *P*3_2_21 is arbitrary.

### Crystal data

**Table:**

C_14_H_19_N_3_O_7_·0.5H_2_O	*D*_x_ = 1.459 Mg m^−^^3^
*M**_r_* = 350.33	Mo *K*α radiation, λ = 0.71073 Å
Trigonal, *P*3_1_21	Cell parameters from 6972 reflections
Hall symbol: P 31 2"	θ = 2.7–27.9°
*a* = 8.6373 (12) Å	µ = 0.12 mm^−^^1^
*c* = 37.021 (7) Å	*T* = 113 K
*V* = 2391.8 (7) Å^3^	Block, colourless
*Z* = 6	0.28 × 0.25 × 0.21 mm
*F*(000) = 1110	

### Data collection

**Table:**

Rigaku Saturn CCD area-detector diffractometer	2242 independent reflections
Radiation source: rotating anode	2161 reflections with *I* > 2σ(*I*)
confocal	*R*_int_ = 0.038
Detector resolution: 7.31 pixels mm^-1^	θ_max_ = 27.9°, θ_min_ = 2.7°
ω and φ scans	*h* = −8→11
Absorption correction: multi-scan (*CrystalClear*; Rigaku/MSC, 2005)	*k* = −11→8
*T*_min_ = 0.967, *T*_max_ = 0.975	*l* = −46→47
15783 measured reflections	

### Refinement

**Table:**

Refinement on *F*^2^	Secondary atom site location: difference Fourier map
Least-squares matrix: full	Hydrogen site location: mixed
*R*[*F*^2^ > 2σ(*F*^2^)] = 0.033	H atoms treated by a mixture of independent and constrained refinement
*wR*(*F*^2^) = 0.085	*w* = 1/[σ^2^(*F*_o_^2^) + (0.034*P*)^2^ + 1.0221*P*] where *P* = (*F*_o_^2^ + 2*F*_c_^2^)/3
*S* = 1.09	(Δ/σ)_max_ = 0.001
2242 reflections	Δρ_max_ = 0.45 e Å^−^^3^
235 parameters	Δρ_min_ = −0.18 e Å^−^^3^
0 restraints	Extinction correction: *SHELXL97* (Sheldrick, 2008)
Primary atom site location: structure-invariant direct methods	Extinction coefficient: 0.0188 (16)

### Special details

**Table:**

Geometry. All e.s.d.'s (except the e.s.d. in the dihedral angle between two l.s. planes) are estimated using the full covariance matrix. The cell e.s.d.'s are taken into account individually in the estimation of e.s.d.'s in distances, angles and torsion angles; correlations between e.s.d.'s in cell parameters are only used when they are defined by crystal symmetry. An approximate (isotropic) treatment of cell e.s.d.'s is used for estimating e.s.d.'s involving l.s. planes.
Refinement. Refinement of *F*^2^ against ALL reflections. The weighted *R*-factor *wR* and goodness of fit *S* are based on *F*^2^, conventional *R*-factors *R* are based on *F*, with *F* set to zero for negative *F*^2^. The threshold expression of *F*^2^ > σ(*F*^2^) is used only for calculating *R*-factors(gt) *etc*. and is not relevant to the choice of reflections for refinement. *R*-factors based on *F*^2^ are statistically about twice as large as those based on *F*, and *R*- factors based on ALL data will be even larger.

### Fractional atomic coordinates and isotropic or equivalent isotropic displacement parameters (Å^2^)

**Table:**

	*x*	*y*	*z*	*U*_iso_*/*U*_eq_	
O1	0.61554 (18)	0.67753 (19)	0.64253 (3)	0.0159 (3)	
O2	0.35880 (19)	0.6142 (2)	0.59033 (4)	0.0214 (3)	
H2	0.3061	0.5826	0.5703	0.032*	
O3	0.6592 (2)	0.4735 (2)	0.55878 (3)	0.0198 (3)	
H3	0.5811	0.4704	0.5448	0.030*	
O4	0.90103 (18)	0.5337 (2)	0.61006 (3)	0.0172 (3)	
H4	0.9643	0.5345	0.6274	0.026*	
O5	0.79887 (18)	0.41000 (19)	0.68003 (3)	0.0174 (3)	
H5	0.7474	0.3599	0.6994	0.026*	
O6	0.67202 (18)	0.64621 (18)	0.70151 (3)	0.0168 (3)	
O7	1.4664 (2)	1.6732 (2)	0.85781 (4)	0.0209 (3)	
O8	0.5415 (3)	1.0000	0.8333	0.0238 (5)	
N1	1.1795 (2)	1.3137 (2)	0.80203 (4)	0.0184 (4)	
N2	1.2921 (3)	1.4869 (2)	0.81373 (5)	0.0223 (4)	
C6	0.5446 (3)	0.7359 (3)	0.58453 (5)	0.0186 (4)	
H6A	0.5749	0.8567	0.5929	0.022*	
H6B	0.5716	0.7429	0.5584	0.022*	
N3	1.3533 (2)	1.3743 (2)	0.86433 (5)	0.0221 (4)	
H3A	1.4048	1.3879	0.8855	0.027*	
H3B	1.2877	1.2666	0.8552	0.027*	
C1	0.6556 (3)	0.6741 (3)	0.60486 (5)	0.0151 (4)	
H1	0.7854	0.7592	0.6005	0.018*	
C2	0.6120 (3)	0.4842 (3)	0.59529 (5)	0.0157 (4)	
H2A	0.4811	0.4004	0.5987	0.019*	
C3	0.7162 (3)	0.4258 (3)	0.61925 (5)	0.0154 (4)	
H3C	0.6766	0.2975	0.6143	0.018*	
C4	0.6833 (3)	0.4471 (3)	0.65893 (5)	0.0147 (4)	
H4A	0.5561	0.3603	0.6652	0.018*	
C5	0.7213 (3)	0.6362 (3)	0.66559 (5)	0.0148 (4)	
H5A	0.8511	0.7235	0.6618	0.018*	
C7	0.7687 (2)	0.8103 (3)	0.71830 (5)	0.0152 (4)	
C8	0.7913 (3)	0.8084 (3)	0.75549 (5)	0.0192 (4)	
H8	0.7354	0.6986	0.7684	0.023*	
C9	0.8963 (3)	0.9682 (3)	0.77356 (5)	0.0194 (4)	
H9	0.9122	0.9672	0.7989	0.023*	
C10	0.9789 (3)	1.1305 (3)	0.75499 (5)	0.0173 (4)	
C11	0.9447 (3)	1.1300 (3)	0.71811 (5)	0.0179 (4)	
H11	0.9930	1.2403	0.7055	0.021*	
C12	0.8411 (3)	0.9710 (3)	0.69954 (5)	0.0172 (4)	
H12	0.8200	0.9721	0.6744	0.021*	
C13	1.1015 (3)	1.3027 (3)	0.77191 (5)	0.0182 (4)	
H13	1.1240	1.4097	0.7601	0.022*	
C14	1.3745 (3)	1.5160 (3)	0.84621 (5)	0.0167 (4)	
H8O	0.555 (5)	0.913 (5)	0.8381 (10)	0.070 (12)*	
H2N	1.304 (4)	1.579 (4)	0.8014 (7)	0.031 (7)*	

### Atomic displacement parameters (Å^2^)

**Table:**

	*U*^11^	*U*^22^	*U*^33^	*U*^12^	*U*^13^	*U*^23^
O1	0.0173 (7)	0.0204 (7)	0.0126 (6)	0.0114 (6)	−0.0008 (5)	−0.0020 (5)
O2	0.0171 (7)	0.0322 (9)	0.0146 (6)	0.0122 (7)	−0.0017 (5)	−0.0015 (6)
O3	0.0229 (8)	0.0301 (8)	0.0120 (6)	0.0174 (7)	−0.0025 (6)	−0.0048 (6)
O4	0.0149 (7)	0.0242 (7)	0.0132 (6)	0.0103 (6)	0.0006 (5)	0.0007 (5)
O5	0.0170 (7)	0.0209 (7)	0.0148 (6)	0.0099 (6)	0.0021 (5)	0.0046 (5)
O6	0.0170 (7)	0.0178 (7)	0.0123 (6)	0.0063 (6)	0.0014 (5)	−0.0022 (5)
O7	0.0250 (8)	0.0192 (7)	0.0170 (6)	0.0100 (6)	−0.0016 (6)	−0.0026 (6)
O8	0.0316 (10)	0.0150 (10)	0.0194 (10)	0.0075 (5)	−0.0008 (4)	−0.0016 (8)
N1	0.0222 (9)	0.0152 (8)	0.0181 (8)	0.0095 (7)	−0.0023 (7)	−0.0023 (6)
N2	0.0326 (10)	0.0155 (9)	0.0187 (8)	0.0121 (8)	−0.0074 (8)	−0.0017 (7)
C6	0.0200 (9)	0.0194 (10)	0.0165 (9)	0.0099 (8)	0.0015 (8)	0.0010 (8)
N3	0.0242 (9)	0.0194 (9)	0.0207 (8)	0.0093 (7)	−0.0056 (7)	0.0018 (7)
C1	0.0155 (9)	0.0172 (9)	0.0120 (8)	0.0077 (8)	0.0014 (7)	−0.0001 (7)
C2	0.0152 (9)	0.0177 (9)	0.0129 (8)	0.0072 (7)	0.0010 (7)	−0.0016 (7)
C3	0.0154 (9)	0.0152 (9)	0.0152 (8)	0.0072 (7)	0.0009 (7)	0.0001 (7)
C4	0.0126 (9)	0.0153 (9)	0.0140 (8)	0.0054 (7)	−0.0001 (7)	0.0005 (7)
C5	0.0137 (8)	0.0181 (9)	0.0119 (8)	0.0074 (7)	0.0010 (7)	−0.0003 (7)
C7	0.0137 (8)	0.0167 (9)	0.0158 (8)	0.0081 (7)	−0.0009 (7)	−0.0025 (7)
C8	0.0234 (10)	0.0182 (9)	0.0162 (9)	0.0106 (8)	0.0002 (7)	0.0020 (7)
C9	0.0258 (10)	0.0218 (10)	0.0126 (8)	0.0133 (9)	−0.0012 (8)	0.0005 (7)
C10	0.0196 (10)	0.0179 (9)	0.0179 (9)	0.0121 (8)	−0.0016 (7)	−0.0020 (7)
C11	0.0204 (10)	0.0174 (9)	0.0175 (9)	0.0106 (8)	−0.0002 (8)	0.0007 (7)
C12	0.0194 (9)	0.0210 (10)	0.0127 (8)	0.0113 (8)	0.0001 (7)	0.0003 (7)
C13	0.0237 (10)	0.0178 (9)	0.0155 (9)	0.0123 (8)	−0.0008 (8)	−0.0001 (7)
C14	0.0183 (9)	0.0185 (10)	0.0148 (8)	0.0104 (8)	0.0006 (7)	−0.0001 (7)

### Geometric parameters (Å, °)

**Table:**

O1—C5	1.419 (2)	N3—H3B	0.8800
O1—C1	1.441 (2)	C1—C2	1.530 (3)
O2—C6	1.428 (2)	C1—H1	1.0000
O2—H2	0.8400	C2—C3	1.518 (3)
O3—C2	1.428 (2)	C2—H2A	1.0000
O3—H3	0.8400	C3—C4	1.524 (2)
O4—C3	1.430 (2)	C3—H3C	1.0000
O4—H4	0.8400	C4—C5	1.517 (3)
O5—C4	1.424 (2)	C4—H4A	1.0000
O5—H5	0.8400	C5—H5A	1.0000
O6—C7	1.381 (2)	C7—C12	1.390 (3)
O6—C5	1.412 (2)	C7—C8	1.392 (2)
O7—C14	1.257 (2)	C8—C9	1.387 (3)
O8—H8O	0.84 (3)	C8—H8	0.9500
N1—C13	1.281 (2)	C9—C10	1.395 (3)
N1—N2	1.385 (2)	C9—H9	0.9500
N2—C14	1.355 (2)	C10—C11	1.397 (3)
N2—H2N	0.88 (3)	C10—C13	1.466 (3)
C6—C1	1.511 (3)	C11—C12	1.390 (3)
C6—H6A	0.9900	C11—H11	0.9500
C6—H6B	0.9900	C12—H12	0.9500
N3—C14	1.326 (3)	C13—H13	0.9500
N3—H3A	0.8800		
			
C5—O1—C1	112.67 (14)	O5—C4—C5	110.79 (15)
C6—O2—H2	109.5	O5—C4—C3	107.90 (15)
C2—O3—H3	109.5	C5—C4—C3	109.50 (15)
C3—O4—H4	109.5	O5—C4—H4A	109.5
C4—O5—H5	109.5	C5—C4—H4A	109.5
C7—O6—C5	116.12 (15)	C3—C4—H4A	109.5
C13—N1—N2	114.29 (17)	O6—C5—O1	107.44 (14)
C14—N2—N1	119.87 (16)	O6—C5—C4	108.02 (15)
C14—N2—H2N	118.8 (18)	O1—C5—C4	110.68 (15)
N1—N2—H2N	121.1 (18)	O6—C5—H5A	110.2
O2—C6—C1	110.08 (16)	O1—C5—H5A	110.2
O2—C6—H6A	109.6	C4—C5—H5A	110.2
C1—C6—H6A	109.6	O6—C7—C12	122.61 (16)
O2—C6—H6B	109.6	O6—C7—C8	116.71 (17)
C1—C6—H6B	109.6	C12—C7—C8	120.69 (18)
H6A—C6—H6B	108.2	C9—C8—C7	119.40 (19)
C14—N3—H3A	120.0	C9—C8—H8	120.3
C14—N3—H3B	120.0	C7—C8—H8	120.3
H3A—N3—H3B	120.0	C8—C9—C10	120.94 (17)
O1—C1—C6	105.85 (14)	C8—C9—H9	119.5
O1—C1—C2	108.28 (15)	C10—C9—H9	119.5
C6—C1—C2	113.83 (16)	C9—C10—C11	118.51 (18)
O1—C1—H1	109.6	C9—C10—C13	123.76 (17)
C6—C1—H1	109.6	C11—C10—C13	117.73 (18)
C2—C1—H1	109.6	C12—C11—C10	121.15 (18)
O3—C2—C3	107.20 (15)	C12—C11—H11	119.4
O3—C2—C1	111.32 (15)	C10—C11—H11	119.4
C3—C2—C1	110.59 (15)	C11—C12—C7	119.07 (17)
O3—C2—H2A	109.2	C11—C12—H12	120.5
C3—C2—H2A	109.2	C7—C12—H12	120.5
C1—C2—H2A	109.2	N1—C13—C10	122.22 (18)
O4—C3—C2	107.37 (15)	N1—C13—H13	118.9
O4—C3—C4	111.43 (15)	C10—C13—H13	118.9
C2—C3—C4	110.23 (15)	O7—C14—N3	123.06 (18)
O4—C3—H3C	109.3	O7—C14—N2	119.55 (17)
C2—C3—H3C	109.3	N3—C14—N2	117.39 (18)
C4—C3—H3C	109.3		
			
C13—N1—N2—C14	176.28 (19)	O5—C4—C5—O6	−67.07 (19)
C5—O1—C1—C6	−175.23 (15)	C3—C4—C5—O6	174.03 (15)
C5—O1—C1—C2	62.35 (19)	O5—C4—C5—O1	175.56 (14)
O2—C6—C1—O1	−62.49 (19)	C3—C4—C5—O1	56.7 (2)
O2—C6—C1—C2	56.3 (2)	C5—O6—C7—C12	31.4 (3)
O1—C1—C2—O3	−176.33 (15)	C5—O6—C7—C8	−148.63 (17)
C6—C1—C2—O3	66.3 (2)	O6—C7—C8—C9	176.08 (18)
O1—C1—C2—C3	−57.27 (19)	C12—C7—C8—C9	−3.9 (3)
C6—C1—C2—C3	−174.68 (15)	C7—C8—C9—C10	0.2 (3)
O3—C2—C3—O4	54.20 (19)	C8—C9—C10—C11	4.0 (3)
C1—C2—C3—O4	−67.32 (18)	C8—C9—C10—C13	−175.52 (19)
O3—C2—C3—C4	175.75 (15)	C9—C10—C11—C12	−4.5 (3)
C1—C2—C3—C4	54.2 (2)	C13—C10—C11—C12	174.99 (19)
O4—C3—C4—O5	−54.48 (19)	C10—C11—C12—C7	0.9 (3)
C2—C3—C4—O5	−173.59 (15)	O6—C7—C12—C11	−176.63 (17)
O4—C3—C4—C5	66.18 (19)	C8—C7—C12—C11	3.4 (3)
C2—C3—C4—C5	−52.9 (2)	N2—N1—C13—C10	179.26 (18)
C7—O6—C5—O1	−91.06 (18)	C9—C10—C13—N1	19.1 (3)
C7—O6—C5—C4	149.50 (15)	C11—C10—C13—N1	−160.4 (2)
C1—O1—C5—O6	179.21 (14)	N1—N2—C14—O7	−174.97 (17)
C1—O1—C5—C4	−63.07 (19)	N1—N2—C14—N3	5.1 (3)

### Hydrogen-bond geometry (Å, °)

**Table:**

*D*—H···*A*	*D*—H	H···*A*	*D*···*A*	*D*—H···*A*
O2—H2···O7^i^	0.84	1.85	2.6905 (19)	175
O3—H3···O8^ii^	0.84	1.94	2.7447 (16)	159
O4—H4···O5^iii^	0.84	2.05	2.8277 (19)	154
O4—H4···O5	0.84	2.34	2.7725 (19)	113
O5—H5···O2^iv^	0.84	1.85	2.693 (2)	178
N3—H3A···O4^v^	0.88	2.24	3.096 (2)	165
N3—H3A···O3^v^	0.88	2.46	2.896 (2)	111
O8—H8O···O7^vi^	0.84 (3)	1.95 (3)	2.7163 (17)	151 (4)
N2—H2N···O3^vii^	0.88 (3)	2.14 (3)	3.014 (2)	176 (2)

Symmetry codes: (i) −*x*+*y*, −*x*+2, *z*−1/3; (ii) −*x*+*y*, −*x*+1, *z*−1/3; (iii) −*x*+2, −*x*+*y*+1, −*z*+4/3; (iv) −*x*+1, −*x*+*y*, −*z*+4/3; (v) −*y*+2, *x*−*y*+1, *z*+1/3; (vi) *x*−1, *y*−1, *z*; (vii) −*x*+2, −*x*+*y*+2, −*z*+4/3.
